# Pro-Apoptotic Effect of Rice Bran Inositol Hexaphosphate (IP_6_) on HT-29 Colorectal Cancer Cells

**DOI:** 10.3390/ijms141223545

**Published:** 2013-12-02

**Authors:** Nurul Husna Shafie, Norhaizan Mohd Esa, Hairuszah Ithnin, Norazalina Saad, Ashok Kumar Pandurangan

**Affiliations:** 1 Laboratory of Molecular Biomedicine, Institute of Bioscience, Universiti Putra Malaysia, Serdang 43400, Selangor, Malaysia; E-Mail: nhusna.shafie@gmail.com; 2 Department of Nutrition and Dietetics, Faculty of Medicine and Health Sciences, Universiti Putra Malaysia, Serdang 43400, Selangor, Malaysia; E-Mail: panduashokkumar@gmail.com; 3 UPM-MAKNA Cancer Research Laboratory, Institute of Bioscience, Universiti Putra Malaysia, Serdang 43400, Selangor, Malaysia; E-Mails: hairuszah@upm.edu.my (H.I.); norazalinasaad@gmail.com (N.S.); 4 Department of Pathology, Faculty of Medicine and Health Sciences, Universiti Putra Malaysia, Serdang 43400, Selangor, Malaysia

**Keywords:** inositol hexaphosphate, rice bran, apoptosis, colorectal cancer

## Abstract

Inositol hexaphosphate (IP_6_), or phytic acid is a natural dietary ingredient and has been described as a “natural cancer fighter”, being an essential component of nutritional diets. The marked anti-cancer effect of IP_6_ has resulted in our quest for an understanding of its mechanism of action. In particular, our data provided strong evidence for the induction of apoptotic cell death, which may be attributable to the up-regulation of *Bax* and down-regulation of *Bcl-xl* in favor of apoptosis. In addition, the up-regulation of caspase-3 and -8 expression and activation of both caspases may also contribute to the apoptotic cell death of human colorectal adenocarcinoma HT-29 cells when exposed to IP_6_. Collectively, this present study has shown that rice bran IP_6_ induces apoptosis, by regulating the pro- and anti-apoptotic markers; *Bax* and *Bcl-xl* and via the activation of caspase molecules (caspase-3 and -8).

## 1. Introduction

Colorectal cancer (CRC) is the third most common cancer in men and the second most common cancer in women worldwide [1]. Despite advances in therapeutic interventions over the past decades, about 40% of the patients will still eventually die because of the disease mainly due to metastasis to the liver [2]. Cancer generally occurs through a multistep sequence of events where the genomes of new tumor cells inherit, alter and/or acquire mutant alleles of oncogenes, tumor suppressor genes, and other genes that control cell proliferation process [3,4] and have abnormal regulation compared to normal cells. Cells lose their normal features and acquire abnormal characteristics that change the morphology of cells, protein expression on the surface of the cell membrane, and the regulatory mechanisms of cell proliferation and death [5].

There is evidence that high consumption of whole grains reduces CRC risk in women [6] and epidemiological studies, pre-clinical and clinical interventions further support this, highlighting the possible protective role that minor components of fiber, other nutrients, and phytochemicals present in wheat bran, grains, and legumes may have against CRC [7]. These include phytic acid or so-called inositol hexaphosphate (IP_6_) which is mainly located in the bran fraction of whole-grain cereals, especially within the aleurone layer. In particular, our study employed rice bran which contains high concentrations of phytic acid ranging from 5.94 to 6.09 g 100 g^−1^[8]. IP_6_ constitutes from 9.5 to 14.5% (*w*/*w*) of the rice bran that has been reported to possess various medicinal properties [9].

Promotion of apoptosis is currently a major goal as a strategy for cancer therapy. Apoptosis-inducing agents may represent a practical mechanistic approach to both cancer chemoprevention and chemotherapy. Exposure to a hormone or growth factor can easily trigger apoptosis in normal and malignant cells. Failure of apoptosis through overexpression of cell survival genes may be involved in the development of many tumors. Additionally, to overcome the initial problems of existing cancer treatments which that also show adverse effects on normal cells, there is a need for a more selective therapy that can directly target the apoptosis machinery of cancer cells only, without affecting normal cells. Many researchers are now focusing on developing novel agents that may enhance the induction of apoptosis in cancer cells. Agents that selectively target mitochondria, which is a major target in early apoptosis induction are being investigated in order to develop tumor selective anti-cancer agents [10]. The Bcl-2 family of proteins in particular, is involved in the control and regulation of apoptotic mitochondrial events [10,11]. Pro-survival Bcl-2 family members control the process of apoptosis by regulating pro-apoptotic Bcl-2 family members, such as Bax and Bak [12]. In general, activation of Bax and Bak involves homo-dimerization and oligomerization within the outer mitochondrial membrane leading to the release of apoptogenic proteins, such as cytochrome c and Smac/DIABLO, from the mitochondrial inter-membrane space [13]. This in turn, promotes activation of the caspase signaling cascade that culminates in proteolysis of hundreds of intra-cellular proteins and consequent cellular destruction. Conversely, both anti-apoptotic proteins, Bcl-2 and Bcl-xl, inhibit the release of cytochrome c from the mitochondria. Furthermore, Newmeyer *et al*. [14] reported that the activation of caspase proteases, which are controlled by anti-apoptotic, Bcl-2 and Bcl-xl lead to the inhibition of apoptotic cell death.

Therefore, agents that can induce apoptosis in cancer cells and spare the normal cells would perhaps enhance the therapeutic profile in combination with chemotherapy or irradiation; *i.e.*, to reduce adverse effects due to apoptosis of normal cells [15,16]. IP_6_ has been shown to inhibit the growth of a wide variety of tumor cells in multiple experimental model systems. The mechanisms underlying the apoptosis induction by IP_6_ seems to be varied and dependent on cell types. Administration with IP_6_ demonstrated no marked toxicity at the optimal doses required for tumor inhibition [17–19]. Furthermore, we found no toxic effects in liver and kidney of rats given IP_6_ (0.2%–0.5% *w*/*v*) in drinking water [20] and likewise no toxicity to the normal 3T3 cell line [21,22].

## 2. Results

### 2.1. IP_6_ Induces Apoptosis and Regulates the mRNA Level of Pro- and Anti-Apoptotic *Bax* and *Bcl-xl* Expression

IP_6_ treatment showed marked growth inhibition and enhanced apoptotic cell death of human colorectal adenocarcinoma HT-29 cells in a dose- and time-dependent manner. Initially, we proved strong evidence that IP_6_ showed an increase of early apoptosis in HT-29 cells using Annexin V assay. IP_6_ elicited its growth inhibitory effects by increasing the total number of apoptosis; 9%, 22% and 41% following 9.5, 12.0 or 14.5 μg/mL of IP_6_, respectively, relative to the control in a dose-dependent manner after 24 h of incubation [21]. Moreover, incubation with IP_6_ (9.5 μg/mL) also markedly increased the total number of apoptoses: 12%, 27% and 38% after 24, 48 or 72 h, respectively, relative to the control in a time-dependent manner [21]. However, the associated signaling pathways and cellular events controlling apoptosis were varied depending on the types of cancer and not well defined. We further examined the molecular mechanism by which IP_6_ induces apoptotic cell death in HT-29 cells at mRNA and protein levels.

Apoptosis can be triggered by both internal and external stimuli, causing deregulated mechanisms in different cancers. Induction of apoptosis is important in cancer treatment. To investigate the molecular mechanism of apoptosis induced by IP_6_ in HT-29 cells, the mRNA expression levels of pro- and anti-apoptotic genes, *Bax* and *Bcl-xl* respectively were examined. The melting curve analysis of the products showed a single peak for each target gene (data not shown). Each gene of interest resulted in an efficiency of 1.9–2.1 (slope of −2.9 to −3.5) over a minimum of three-log concentration range. Furthermore, the similar amplification efficiency of gene of interests with housekeeping genes was confirmed by assuring a slope of less than 0.1 when ΔC_T_ (the difference between target gene C_T_ and housekeeping gene C_T_) was plotted against log concentration of RNA (data was not shown).

**Figure 1.**
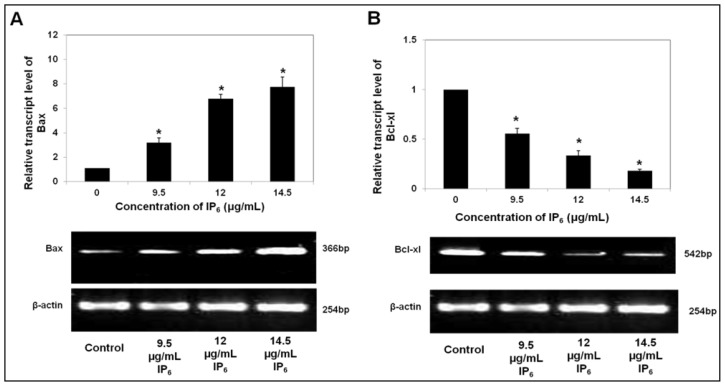
Expression of apoptotic related genes at mRNA level in HT-29 cells incubated with IP_6_. (**A**) Upregulation of pro-apoptotic, *Bax*; and (**B**) Downregulation of anti-apoptotic, *Bcl-xl* in a dose-dependent manner. HT-29 cells were incubated with IP_6_ (9.5; 12 or 14.5 μg/mL) for 72 h at 37 °C. Results are from 3 independent experiments and presented as the mean ± SD. * indicate significant difference by Tukey’s test (*p* < 0.05) relative to their respective control.

Additionally, 5% agarose gel electrophoresis of the PCR product exhibited the presence of a single product band and the absence of a primer dimer band. The change in mRNA expression was analyzed using ΔΔC_T_ method with β-actin as housekeeping gene. Figure 1 showed that IP_6_ treatment of cells at various doses (9.5, 12.0 or 14.5 μg/mL) for 72 h marked a noticeable alteration in *Bax* and *Bcl-xl* mRNA level compared to control (untreated HT-29 cells) (*p* < 0.05). Notably, IP_6_ upregulated the transcript of the pro-apoptotic gene, *Bax* and downregulated the transcription of the anti-apoptotic gene, *Bcl-xl* by several fold. The expression level of *Bax* in HT-29 cells incubated with 9.5, 12.0 or 14.5 μg/mL of IP_6_ for 72 h increased by 3.18, 6.78 or 7.76 fold respectively as compared with control (Figure 1A). In contrast, the expression level of *Bcl-xl* in HT-29 cells incubated with 9.5, 12.0 or 14.5 μg/mL of IP_6_ for 72 h reduced by 0.56, 0.34 or 0.18 fold, respectively (Figure 1B). Collectively, these results show a marked increase in *Bax* and decrease in *Bcl-xl* expression that were found to be dose-dependent.

### 2.2. IP_6_ Upregulates Caspase Gene Expression

**Figure 2.**
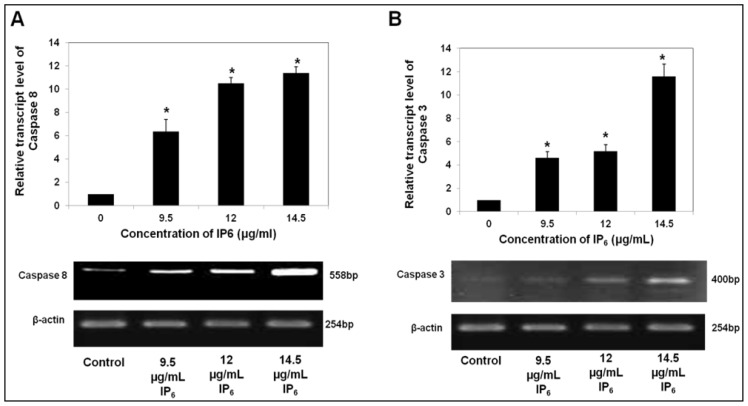
Expression of caspases at mRNA level of HT-29 cells incubated with IP_6_ (**A**) upregulation of *caspase-8* and (**B**) upregulation of *caspase-3* in a dose-dependent manner. HT-29 cells were incubated with IP_6_ (9.5, 12.0 or 14.5 μg/mL) for 72 h at 37 °C. Results are from 3 independent experiments and presented as the mean ± SD. * indicate significant difference by Tukey’s test (*p* < 0.05) relative to their respective control.

Caspase-8 is an initiator, while caspase-3 is an executioner, where activation of both caspases represent important biochemical markers that have been identified in cells favoring apoptosis [23]. Figure 2 summarized the gene expression changes of *caspase-8* and *-3*. It was clearly shown that IP_6_ upregulated the transcript of *caspase-8* and *-3* at mRNA level by several folds. The expression levels of *caspase-8* gene in HT-29 cells treated with 9.5, 12.0 or 14.5 μg/mL of IP_6_ for 72 h were significantly (*p* < 0.05) increased by 6.38, 10.5 or 11.4 fold, respectively, as compared to the levels in control (Figure 2A). Similarly, the expression levels of *caspase-3* gene in HT-29 cells incubated with 9.5, 12.0 or 14.5 μg/mL of IP_6_ for 72 h increased by 4.63, 5.17 or 11.6 fold, respectively as compared to the levels in control (Figure 2B). Together, these data suggest that IP_6_ caused a marked increase in apoptosis, which was accompanied by upregulation of caspase-8 and caspase-3 expression at mRNA level.

### 2.3. IP_6_ Modulates Apoptosis Related Protein Expression, *Bax* and *Bcl-xl*

**Figure 3.**
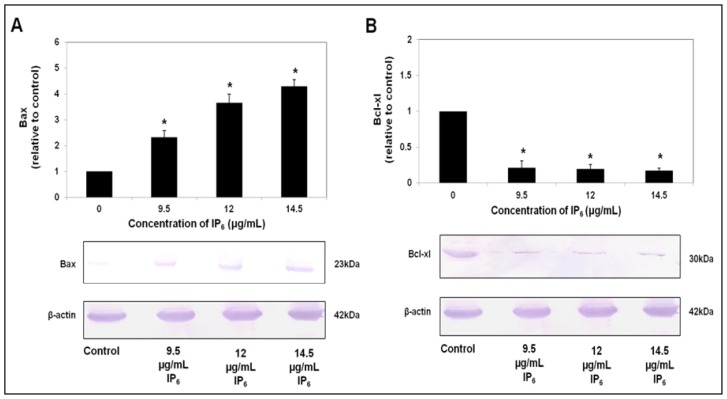
The effect of IP_6_ on apoptotic protein levels in HT-29 cells. (**A**) IP_6_ increased protein expression of Bax; (**B**) IP_6_ reduced protein expression of Bcl-xl in a dose-dependent manner. Results are from 3 independent experiments and presented as the mean ± SD. * indicate significant difference by Tukey’s test (*p* < 0.05) relative to their respective control.

In order to establish that the changes in mRNA levels monitored by RT-PCR were reflecting changes at the protein level and for further confirmation for translation of apoptosis related genes’ mRNAs into apoptosis related proteins, western blot analysis was performed in parallel. As mentioned previously, Bax was upregulated and Bcl-xl was downregulated at the mRNA level after incubation with IP_6_ in HT-29 cells as shown in Figure 1. Likely, similar changes can be observed at the protein level as well. Pro-apoptotic, Bax protein level, which was detected as a 23 kDa band, was significantly (*p* < 0.05) increased after IP_6_ treatment of HT-29 cells in a dose-dependent manner (Figure 3A). The protein expression level of Bax of HT-29 after incubation with 9.5, 12.0 or 14.5 μg/mL for 72 h was increased by 2.33, 3.65 or 4.3 fold respective to the control. Furthermore, Bcl-xl which was detected as a 30 kDa band was reduced by 0.21, 0.19 or 0.17 fold after incubation with 9.5, 12.0 or 14.5 μg/mL of IP_6_, respectively, relative to the control (Figure 3B).

### 2.4. IP_6_ Activates Caspase-8 and -3

**Figure 4.**
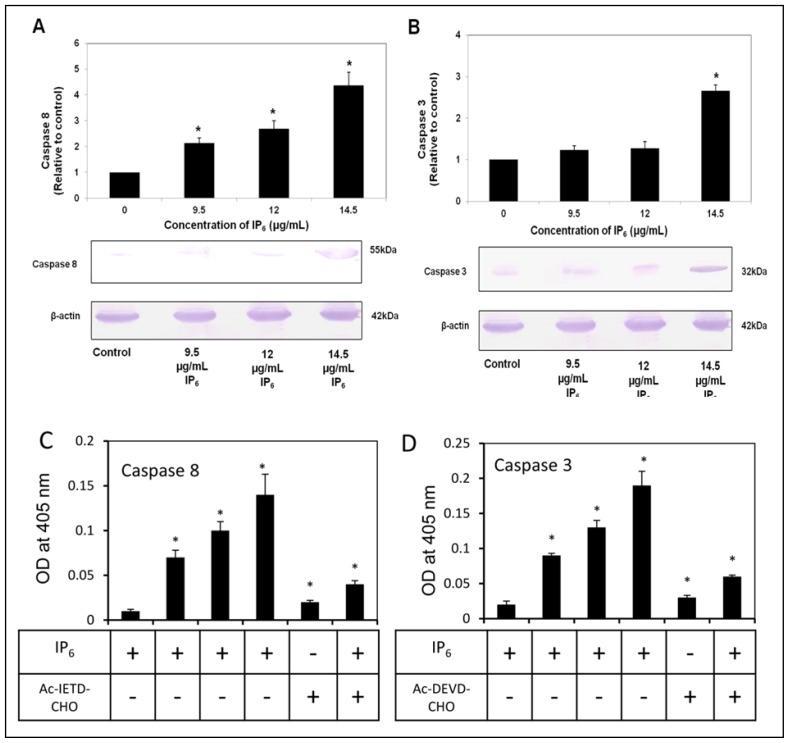
The effect of IP_6_ on caspase protein levels in HT-29 cells (**A**) IP_6_ increased protein expression of caspase-8 in a dose dependent manner (9.5; 12.0 or 14.5 μg/mL of IP_6_); (**B**) IP_6_ increased the expression of caspase-3 at highest dose of IP_6_ (14.5 μg/mL); (**C** and **D**) The caspase-8 and -3 activities were determined by the incubation with specific substrates Ac-IETD-pNA (for caspase 8) and Ac-DEVD-pNA (for caspase 3); respectively. Subsequently addition of inhibitors of caspase 8 (Ac-IETD-CHO) and 3 (Ac-DEVD-CHO) shows reduction in the activities were observed. The combination of inhibitor and IP_6_ showed diminished activities of caspase 8 and 3 were observed. The optical density was measured at 405 nm as described in materials and methods section. Results are from 3 independent experiments and presented as the mean ± SD. * and * indicate significant difference by Tukey’s test (*p* < 0.05) relative to their respective control.

To examine the translational confirmation of caspase-8 and -3, western blot analysis for protein assay was performed. Interestingly, caspase-8 and -3 protein expression, detected as 55 and 32 kDa bands respectively, were markedly increased after IP_6_ treatment of HT-29 cells in a dose-dependent manner as compared to the control (Figure 4A,B). We clearly observed that both caspases were significantly (*p* < 0.05) upregulated by 2.13, 2.7 or 4.37 fold for caspase-8 and 1.24, 1.28 or 2.66 fold for caspase-3 relative to their respective control following the IP_6_ treatments (9.5; 12.0 or 14.5 μg/mL). The results showed that the increased caspase-8 was observed in a dose-dependent manner (Figure 4A). Notably, IP_6_ has shown significantly (*p* < 0.05) increased caspase-3 protein expression only after incubation with the highest dose of IP_6_, 14.5 μg/mL (Figure 4B). The activation of caspase protein content was due to the proteolytic cleavage of caspases occurred during apoptosis because these changes were observed after the induction of apoptosis, which in this case triggered after 72 h of IP_6_ treatment. To further confirm the enzymatic activity of caspase-8 and -3, the colorimetric measurement of respective substrates of caspases were performed. HT-29 cells were incubated with IP_6_ for 72 h at the concentrations of 9.5, 12.0 or 14.5 μg/mL. Notably, we observed a significant (*p* < 0.05) increase in the activities of both caspase-8 and -3 following the increment of the dose of IP_6_, demonstrating a dose-dependent manner (Figure 4C,D). Subsequently, addition of inhibitors of caspase-8 and -3 showed reduced activities were observed. The combination of inhibitor and IP_6_ showed diminished activities of caspase-8 and -3. Collectively, these results show an interaction between changes of mRNA and protein levels of caspases after incubation of IP_6_ in HT-29 cells and activation of caspases were induced by IP_6_ as we clearly observed the increment of initiator caspase-8 and executioner caspase-3.

## 3. Discussion

With regards to *Bax*/*Bcl-xl* ratio in apoptosis process, *Bax* supports apoptosis whereas *Bcl-xl* is an anti-apoptotic molecule. Therefore, we quantified the expression level of pro-apoptotic and anti-apoptotic genes before and after incubation with IP_6_ in HT-29 cells through quantitative RT-PCR and western blot analysis. Apoptotic induction effect of IP_6_ on HT-29 cells was observed after 72 h incubation time whereby *Bcl-xl* expression was inhibited while *Bax* expression was markedly increased in a dose dependent manner. Therefore, it can be implied that apoptosis induced by IP_6_ may be mediated by the *Bax* and *Bcl-xl* in HT-29 cells.

Furthermore, these data show a correlation of changes in both mRNA and protein levels of Bax, Bcl-xl, caspase-3 and -8 after incubation with IP_6_. The downregulation in the expression of Bcl-xl, and the up-regulation in Bax expression at protein level may cause the collapse of mitochondrial membrane potential (ΔΨ*m*), resulting in the release of cytochrome c thus causing apoptosis [24]. Moreover, there is data indicating that overexpression of Bax alone can disrupt mitochondrial membrane integrity and also that formation of the mitochondrial permeability transition (MPT) pore [25,26] can occur, resulting in the release of cytochrome c, a situation that favors apoptosis.

It is interesting to note here that Bax has been shown to promote caspase activation by its effects on mitochondria. This pro-apoptotic *Bcl-2* family member induces the release of proteins from the space between the inner and outer mitochondrial membranes [14]. This process of mitochondrial outer membrane permeabilization (MOMP) results in the release of cytochrome c and other soluble proteins into the cytosol. Therefore, additional studies are needed to define whether IP_6_-mediated alteration in *Bax* and *Bcl-xl* levels activate mitochondrial damage, leading to cytochrome c release and hence activate caspase in its overall apoptotic response in HT-29 cells.

An increasing number of caspases, also known as cysteine proteases that specifically cleave proteins after Asp residues, are absolutely required for the accurate and limited proteolytic events that typify programmed cell death [27]. Thus, it is reasonable to think that caspase activation must play a role in the apoptotic process in HT-29 cells after incubation with IP_6_. This was proven by data gathered from quantitative real-time PCR and western blot analysis, showing that rice bran IP_6_ promoted the levels of caspase-3 and -8.

The current data showed a significant increase in the caspase-3 activity in HT-29 cells after IP_6_ treatment. This agrees with published data by Sharma *et al*. [28], who found that IP_6_ has been shown to significantly increase caspase-3 activity in experimental mouse prostate cancer model. Furthermore, Schroterova *et al*., also reported both IP_6_ and inositol in combination increase the caspase-3 activity on colorectal carcinoma human cell lines HT-29, SW-480 and SW-620 in a time-dependent manner enhancing the proapoptotic effect of IP_6_[29]. Caspase-3 is activated in the apoptotic cell both by extrinsic (death ligand) and intrinsic (mitochondrial) pathways [30]. In intrinsic activation, the up-regulation of Bax by IP_6_ in HT-29 cells may trigger cytochrome c release from the mitochondria in combination with caspase-9, apoptosis-activating factor 1 (Apaf-1) and adenosine triphosphate (ATP) to process procaspase-3 which then activates caspase-3 [31,32]. Therefore, it can be suggested that IP_6_ induced apoptosis by caspase-3 activation was mediated by Bax.

Natural compounds have the ability to induce apoptosis by modulating the expression of caspase-3 [33]. The activation of caspase-3 induced by IP_6_ in HT-29 cells also suggested that an alternative pathway of inducing apoptosis might have been activated. The second extrinsic pathway or cell death receptor pathway is mediated distinctively by active caspase-8 that is characterized by binding cell death ligand and cell death receptors followed by activation of caspase-8 and caspase-3 [16] for apoptosis to occur. As mentioned earlier, besides the up-regulation of caspase-3, this present data also showed a significant increase of caspase-8 after treatment with IP_6_ in HT-29 cells. This can also suggest that the increase of caspase-8 led to subsequent activation of downstream caspase-3 (an apoptotic executioner) which then stimulated the molecular cascade of apoptosis in HT-29 cells.

Moreover, the activated caspase-8 activates caspase-3 through two pathways. In the first pathway, caspase-8 cleaves BID (Bcl2 Interacting Protein) and its carboxyl (COOH)-terminal part translocates to mitochondria where it triggers cytochrome c release then activates a caspase signalling cascade which triggers apoptosis through caspase-3 activation. Another pathway is that caspase-8 cleaves procaspase-3 directly and activates it [34]. However, further analyses are needed to determine which pathway triggers caspase-3 activation by IP_6_. Recently we showed IP6 reduced the tumor number by modulating the wnt/β-catenin pathway during azoxymethane-induced colon cancer in rats [35]. With current data, it can only be suggested that IP_6_ induced caspase-3 activation may be mediated by activation of caspase-8. Therefore, taken together, the data presented in this study suggest that IP_6_-induced apoptosis are mediated by the death receptor and mitochondrial apoptotic pathways as demonstrated by increased expression levels of initiator caspase-8 followed by upregulation of caspase-3 and in accordance with increased and decreased expression level of Bax and Bcl-xl respectively, after IP_6_ treatment.

## 4. Experimental Sections

### 4.1. Chemicals

TRI reagent, 1% agarose gel, tris-borate-EDTA (TBE) buffer and specific primers were purchased from Sigma (St. Louis, MO, USA). Gene Amp Gold RNA PCR Core Kit was bought from Applied Biosystems (Foster City, CA, USA). Agarose gel electrophoresis materials were purchased from 1st Base, Kuala Lumpur, Malaysia. Kapa Sybr Fast qPCR kit (Kapa Biosystems, Boston, MA, USA), AllPrep DNA/RNA/Protein Mini Kit and QIAshredder homogenizer were bought from Qiagen (Dusseldorf, Germany). Western blotting reagents were purchased from BioRAD (Hercules, CA, USA). Dimethylformamide (DMF) was purchased from Fermentas (Vilnius, Lithuania).

### 4.2. Sample Preparation

Freshly milled raw rice bran samples from mixed local varieties were kindly supplied by the BERNAS Milling Plant (Selangor, Malaysia). Extraction and isolation of IP_6_ was initially carried out according to the established methods in our laboratory [20,36].

### 4.3. Cell Culture

Cell lines used in this study, HT-29 (human colorectal cancer cell line) was bought from American Type Culture Collection (ATCC) (Manassas, VA, USA) and the cells were grown in DMEM media with the following supplements; 10% (*v*/*v*) fetal bovine serum (FBS), 100 IU/mL penicillin and 100 μg/mL streptomycin. Cells were grown in sterile cell culture flask at 37 °C in the presence of 5% carbon dioxide (CO_2_). All steps were performed aseptically in a biosafety hood using sterile equipment.

### 4.4. Quantification of Apoptosis Related Genes by Real-Time PCR

HT-29 cells were pre-incubated at a density of 1 × 10^5^ cells in 25 cm^2^ culture flasks for 24 h. The culture medium was replaced with fresh aliquots containing IP_6_ compounds at three different concentrations based on the IC_50_ values: (9.5, 12.0 or 14.5 μg/mL) that were initially carried out in our laboratory [21] and incubated for another 72 h. TRI-reagent (Sigma, St. Louis, MO, USA) was used to extract total RNA directly from cultured cells, according to the manufacturer’s instructions. Then 1 μg of the total RNA sample was reverse transcribed using the Gene Amp Gold RNA PCR Core Kit (Applied Biosystem, Foster, CA, USA) in a final reaction volume of 20 μL, according to the manufacturer’s protocol. The reverse transcription reaction was carried out at 42 °C for 30 min in an authorized thermal cycler (Eppendorf, Hamburg, NY, USA), followed by a 10 min step at 99 °C to denature the enzyme and then it was cooled to 4 °C.

The primers used are listed in Table 1, where the nucleotide numbers indicate the primer location in the corresponding sequences of human origin obtained from National Center for Biotechnology Information Gene Bank. The specific primers were subsequently validated for the specificity of amplification, amplification efficiency over a concentration range and consistency with amplification efficiency of housekeeping gene. All primers used for qRT-PCR were commercially obtained from Sigma (St. Louis, MO, USA). The mRNA levels of *Bax*, *Bcl-xl*, *Caspase-8* and *Caspase-3* were assayed using Kapa Sybr Green Fast qPCR kit (Kapa Biosystems, Boston, MA, USA) and was performed in a reaction volume of 20 μL, according to the manufacturer’s instructions. Real-time PCR was run according to the following conditions: (i) PCR activation at 95 °C for 20 s; (ii) denaturation at 95 °C for 3 s and (iii) annealing/extension at 55–60 °C for 20 s. All samples and controls were run in triplicate on a Mastercycler realplex system (Eppendorf, Hamburg, NY, USA). The quantitative RT-PCR data was analyzed using a comparative threshold (C_T_) method, and the fold inductions of the samples were compared with the untreated samples. *β-actin* was used as an internal reference gene to normalize the expression of the target genes.

### 4.5. Agarose Gel Electrophoresis

Agarose gel (1% (*w*/*v*)) was prepared by adding 0.5 g of agarose to 50 mL of 1× TBE buffer (121.1 g/L Tris Base, 55.0 g/L Boric acid, 500 mM of EDTA (pH 8)). 10 μL of each sample was mixed with 1.0 μL of loading dye [10 mM Tris-HCl (pH 7.6), 0.03% bromophenol blue, 0.03% xylene cyanol, 60% glycerol, 60 mM EDTA]. 10 μL of the appropriate marker was loaded into the first well, and the electrophoresis unit was run for 1 h at 120 V and 300 mA. The gel was stained in 0.2 μg/L of ethidium bromide solution for 20 min and destained in distilled water for 5 min. The gel was viewed and photographed under transluminent UV light in a chemiluminescence imager at 302 nm wavelength.

### 4.6. Western Blot Analysis

Briefly, HT-29 cells were grown in a monolayer in cell culture flasks for 24 h. The culture medium was replaced with fresh aliquots containing IP_6_ compounds at three different concentrations; (9.5, 12.0 or 14.5 μg/mL) and incubated for another 72 h. After treatment, cells were collected and transferred to a new RNase-free tube and centrifuged at 1500 rpm for 5 min. Then extraction of total protein from human cells was performed using AllPrep DNA/RNA/Protein Mini Kit according to the manufacturer’s protocols (Qiagen, Duesseldorf, Germany). Protein concentration was determined by the Bradford assay, according to the manufacturer’s protocol (BioRAD, Berkeley, CA, USA). The protein (50 μg) was separated by 12% sodium dodecyl sulfate polyacrylamide gel electrophoresis (SDS-PAGE) and transferred onto a piece of PVDF membrane using transfer buffer (25 mM Tris-base, 190 mM glycine, 20% (*v*/*v*) methanol; pH 8.3). After transfer, the PVDF membrane was blocked at room temperature with blocking solution (25 mM Tris-base, 0.3 M NaCl, 5% Milk Diluent) (BioRAD, Berkeley, CA, USA) for 30 min. After blocking, the membrane was incubated for overnight with primary antibodies, followed by 2 h with secondary antibodies in Tris-buffered saline (TBS) and 0.5% Tween. Mouse anti-human Bax, Bcl-xl, caspase-8, caspase-3 and β-actin antibodies (Santa Cruz Biotechnology, Dallas, TX, USA) were used at a 1:1000 dilution as the primary antibodies, while alkaline phosphatase-labeled goat anti-mouse antibody (Santa Cruz Biotechnology, Dallas, TX, USA) was used at a 1:10,000 dilution as secondary antibody. The membrane was then exposed and protein bands were detected using developing solution for alkaline phosphatase conjugated antibodies consisted of 10 mL alkaline phosphatase buffer (100 mM Tris-HCl, 100 mM NaCl, 5 mM MgCl_2_; pH 9.5), 33 μL BCIP (0.5 g 5-bromo-4-chloro-3-indolylphosphate-p-toluidine salt (Fermentas, Lithuania, EU) in 10 mL of 100% (*v*/*v*) dimethylformamide (DMF)), and 66 μL NBT (0.75 g nitroblue tetrazolium chloride (Fermentas, Lithuania, EU) in 10 mL of 70% (*v*/*v*) DMF). The reaction was stopped when the desired protein band appeared. Densitometric analysis of band intensities obtained from Western blotting experiments were carried out using ImageJ Software (National Institute of Health, NIH, Bethesda, MD, USA).

### 4.7. Measurement of Caspase-3 and 8 Activities

The protease activity of caspases-3 and 8, in HT-29 cells, was assessed using a colorimetric assay kit (Sigma Aldrich, St. Louis, MO, USA) based on spectrophotometric detection of the caspase enzymes after cleavage from the labeled substrate. About 3 × 10^6^ HT-29 cells were treated with IP_6_ at the concentrations of (9.5, 12.0 or 14.5 μg/mL) and incubated for 72 h. Then, the cells were centrifuged for 5 min at 2000 rpm to remove the media. The cells were then washed two times with PBS and centrifuged at 2000 rpm for 5 min. The cell pellets were lysed by the addition of 50 μL cold prepared lysis buffer containing 0.5 μL DTT and 0.25 μL PMSF, mixed well, and incubated on ice for exactly 1 h. During this time, tubes were vortexed with vibration 3–4 times for 10 s each time. The resulting cell lysate was centrifuged for 1 min at 10,000 rpm at 4 °C, and the supernatant was collected. Briefly, the reaction mixture (total volume, 100 μL) containing 30 μL of cell lysate and 10 μL of the acetyl-Ile-Glu-Thr-Asp-p-nitroaniline (caspase 8 substrate) and acetyl-Asp-Glu-Val-Asp-p-nitroanilide (caspae 3 substrate) (final concentration, 200 μM) in assay buffer, and the assay was carried out in a 96-well plate. The mixtures were incubated for 90 min at 37 °C and the absorbance was read at 405 nm using a Universal Microplate Reader (Bio-TEK. Instrument. Inc., Winooski, VT, USA).

### 4.8. Statistical Analysis

Data were expressed as the mean ± standard deviation (SD) and statistically analyzed using SPSS version 20 (SPSS Inc., Chicago, IL, USA), with a one-way ANOVA with Tukey’s test [37,38] and a significance level of *p* < 0.05.

## 5. Conclusions

We clearly showed that rice bran IP_6_ induces apoptosis, by regulating pro- and anti-apoptotic Bax and Bcl-xl, as well as through the activation of caspase-3 and -8. These results provide molecular evidence for how IP_6_ may elicit the growth inhibition of colon cancer *in vitro*. Hence, we suggest that inositol hexaphosphate (IP_6_), might be useful as a potential preventive and/or therapeutic agent in colon cancer either alone or as an adjunct to standard chemotherapeutic modalities. Moreover, in the future rice bran that is normally discarded might increase in value due to its phytonutrient, IP_6_ potential as nutraceutical products.

## Figures and Tables

**Table 1.** Nucleotide sequence for PCR primers for amplification and sequence-specific detection of cDNA.

Pair No	Primer Name	Accession No	Sequence Location	Oligonucleotides (5′–3′) Sequence
1	*Bax*	L22473	nt 172–195	F-AAGCTGAGCGAGTGTCTCAAGCGC
			nt 516–537	R-TCCCGCCACAAAGATGGTCACG
2	*Bcl-xL*	Z23115	nt 381–402	F-ATGGCAGCAGTAAAGCAAGCGC
			nt 903–922	R-TTCTCCTGGTGGCAATGGCG
3	Caspase-3	U26943	nt 340–361	F-TTTGTTTGTGTGCTTCTGAGCC
			nt 720–739	R-ATTCTGTTGCCACCTTTCGG
4	Caspase-8	X98172	nt 1064–1087	F-GGGACAGGAATGGAACACACTTGG
			nt 1597–1621	R-TCAGGATGGTGAGAATATCATCGCC
5	β-actin	X00351	nt 936–955	F-CTGTCTGGCGGCACCACCAT
			nt 1170–1189	R-GCAACTAAGTCATAGTCCGC
